# Gender differences, academic patenting, and tenure-track reform in China: Evidence from life sciences at elite universities

**DOI:** 10.1371/journal.pone.0307165

**Published:** 2024-07-16

**Authors:** Xin Zhang, Shi Chen, Wei Zheng

**Affiliations:** 1 College of Law, Southwest University of Finance and Economics, Chengdu, Sichuan, China; 2 Carsgen Therapeutics, Shanghai, China; Ingenio CSIC-UPV, SPAIN

## Abstract

This study is the first to examine the gender gap in academic patenting among faculty members in Chinese universities, a critical issue for the sustainable development of scientific research and innovation. Using a unique dataset that includes the patenting activities and professional status of 1,836 faculty members in life science-related departments at 36 top Chinese universities, this research reveals an evolving landscape of patenting dynamics. The trend of male faculty members leading in the annual number of patent applications and patents granted has shifted among newly graduated faculty members. Female faculty submit and receive their first patent applications significantly earlier than male faculty. However, male faculty are more likely to be lead inventors, and this gender gap remains difficult to close, with female faculty more likely to be supporting inventors. This research is contextualized within the broader framework of China’s university tenure reform and the growing presence of women in the life sciences. While progress is evident, the study uncovers persistent systemic barriers that prevent women from fully translating their research into patentable innovations. By identifying these social and institutional barriers, our study not only sheds light on the gender gap, but also suggests policy measures to promote gender equity in scientific innovation, making it a critical read for policymakers and academic leaders.

## Introduction

Gender equality has long been an issue of concern to the academic community [1], as it is important in fostering diversity, collaboration, and equitable access for research innovation and sustainable solutions to global challenges [2]. But academia is found to exhibit a persistent gender gap [2], which is particularly reflected in four different dimensions: research productivity, research impact, rewards, and professional standing [3–7], where female researchers consistently lag behind their male counterparts. Until recently, attention has been paid to the gender gap in the commercialization of research, particularly in patenting [8–11]. This gender gap has not only been interpreted in terms of individual choices and preferences, but also reflects deeper issues within workplace and societal systems [12]. Previous research has shown that the gender gap is influenced to varying degrees by several factors: differences in educational and employment opportunities and career progression between the sexes [13], differences in the resources associated with personal choices [14], differences in the ways in which they build connections and collaborations, and the challenges of balancing work and family responsibilities [15].

To empower female scientists in innovation, the Chinese government has initiated measures to improve their access to resources, increase their decision-making roles, and foster a supportive research environment [16]. Currently, female professionals account for 40% of the science and technology workforce in China [17], which is higher than the global average [2]. Gender gaps in the representation of female researchers are reported to be narrowing in various academic fields, particularly in the life sciences and medicine [17]. However, gender disparities in the quantity and quality of publications and the undertaking of research projects still widely exist [18–20]. Although existing research has examined the relationship between patenting and variables such as gender dominance in certain fields [21], gender diversity in teams [22], and gender composition [23], the gender differences in knowledge transfer and technology commercialization remain understudied.

With China’s ambition to shift to an innovation-driven development strategy for sustainable growth, the government has significantly increased funding to strengthen research capacity and innovation in the higher education sector, resulting in a significant increase in scientific output. Notably, as per the Nature Index Annual Tables, in 2022, China surpasses the United States for the first time in science in the number of published research papers and patents [24]. On the other hand, since the mid-2000s, China has been piloting personnel management reforms in its elite universities, restructuring career paths for both existing and new faculty members. These reforms are seen as an adoption of the American tenure-track system and aim to break up the traditional “iron rice bowl” system that guaranteed lifetime job security for faculty. The introduction of a more transparent, fair, and competitive “merit-based” evaluation and promotion system has led to a significant increase in academic productivity [24].

In this context, patents have become an important measure of innovation efficiency for organizations and academic productivity for individual faculty members. Between 2012 and 2021, university patent applications increased from 106,000 to 367,000, while granted patents increased from 69,000 to 308,000 [25]. Academic institutions now hold for 25.3% of the total number of valid invention patents in China [26]. Therefore, exploring the relationship between academic patenting and gender is an increasingly important and intriguing question.

To determine whether there is a difference in patenting activity between male and female faculty members at Chinese universities, we’ve developed a new dataset. This dataset includes information on patenting and professional status of faculty members from life science-related departments at top Chinese universities. Unlike the patent-level data typically used in most studies in this area, our dataset provides a clearer view of the patenting activities and career trajectories of individual researchers. This study aims to answer three main questions: First, is there a gender gap in the annual number of patent applications filed and received by male and female faculty members? Second, do the contributions to the patents filed differ by gender? Third, are there gender differences in the timing of filing and receiving these patents? If differences in these aspects are found, we plan to analyze how these differences have changed over time and examine possible causes, in particular the influence of tenure-track reforms in Chinese universities on these differences.

To the best of our knowledge, this is the first quantitative study to examine gender disparities among Chinese university researchers in terms of knowledge transfer and commercialization. The issues addressed go beyond the realm of gender equality in science from a sociological perspective to include the strategic management of female scientific talent. This research aims to inform policymakers and academic institutions on how to better address these challenges, promote gender equality, and more effectively commercialize scientific discoveries.

## Literature review

Previous research using large patent databases has consistently found notable gender differences in the proportion of patent applicants in different fields over time [27–29]. Women are reported to be underrepresented in the number of patent applications and the percentage of female inventors [30, 31], to be less likely to have their patent applications approved and maintained [32], although some studies have found that women’s share of patents has been gradually increasing, with rates varying by country [10].

Women’s disadvantages are also evident in the authorship of publications. Research has shown that in several medical subfields, women are less likely to be represented as first or last authors, who are recognized as the primary contributors and project leaders of publications [33]. In cardiovascular and life sciences, women are more likely to hold first author positions, but these positions are predominantly in lower impact journals. Additionally, it has been found that women’s roles as first authors early in their careers rarely translate into senior author positions later on, indicating a deep and consistent gender gap at higher levels of authorship [34]. Regarding patenting, there is limited research on gender differences in inventor rank. One of the few studies in this area found that female inventors are significantly less likely than their male counterparts to be listed as first inventors, which is similar to findings in academic publications [30].

Studies specific to the life sciences, a discipline with a relatively high representation of women, have shown mixed results regarding gender gaps. [32] highlighted that the gender gap in patenting is more pronounced in the life sciences than in other fields, with all-female teams having lower patent approval rates and female inventors receiving fewer prior citations than all-male teams. However, [35] found that despite the lower number of patents filed by women in the life sciences, the quality and impact of their commercial results are comparable to or exceed those of male scientists.

Women’s disadvantages in science and academia are often attributed to their underrepresentation in relevant fields due to limited educational and academic career opportunities [36]. Other contributing factors include gender stereotypes in the workplace [37], family and partner influences [38], and the potential impact of motherhood on career advancement, as identified in case studies using interview surveys [15]. Studies using longitudinal data that track the career paths of doctoral graduates have highlighted the disadvantages faced by female researchers in their early career development as a major contributor to the gender gap in science. Major life events, such as getting married and having children, may contribute to women’s dropout rates from graduate training to tenure [39]. Nearly half of women scientists in the U.S. leave full-time academic positions after the birth of their first child [40], with higher dropout rates observed from the postdoctoral to principal investigator stage [40]. Additionally, female researchers may be underrepresented in interdisciplinary research [41], less likely to achieve high levels of performance [42], and receive less support and fewer resources [43] in the early stages of their careers. These challenges collectively contribute to the persistent gender gap in the scientific and academic fields.

Networks and collaborations are critical to advancing gender equity among academic scientists [44]. Women scientists’ participation in these networks has led to improved career outcomes and increased scholarly output [45]. However, many studies have found gender differences in collaboration. For example, [46] found that women were less likely to participate in university-industry collaborations. [47] reported that women, on average, have more collaborators than men, but men and women choose collaborators differently. Men tend to choose collaborators who can provide practical help or valuable experience, while women often choose collaborators who can provide guidance and support. Additionally, [48, 49] found that female scientists have higher rates of intra-institutional and national collaborations, while male scientists have more international collaborations. Gender diversity in teams significantly improves the collaborative process [50]. Furthermore, [51] found that women face fewer disadvantages in the field of nanotechnology compared to the broader technology field, likely due to the prevalence of interdisciplinary collaboration in this field, which provides women with more opportunities to participate in patent applications.

In China, considerable attention has been paid to evaluating the outcomes and impacts of personnel management reforms in higher education. However, most of the literature has focused on academic output, with little research addressing the impact of these reforms on academic gender inequality. These personnel reforms are primarily seen as an effective response to increasing global competition and a way to improve research performance [30]. For individual faculty members, the system introduced clear and consistent metrics for evaluating performance, focusing on their research output, teaching quality, and community service [52, 53]. In this highly competitive “publish or perish” environment, faculty members must demonstrate substantial scholarly achievement and the potential for continued contributions to their field before securing more permanent, long-term positions. This has led to increased academic productivity and innovation [54]. However, the reform has also been criticized for increasing pressure and anxiety [55], with some arguing that it favors quantity over quality in research [56]. Additionally, there are concerns that it may encourage academic misconduct [57]. Given these issues, it is reasonable to hypothesize that by providing a clearer path for career advancement, bias and favoritism could be reduced, potentially addressing some of the concerns related to gender inequality in academia.

## Data and methods

### Data

Two datasets were compiled and integrated to create the final dataset for analysis. First, faculty lists were compiled from life science departments within the 36 Double First-Class universities. Information such as gender, department affiliation, institutional affiliation, academic title, highest degree earned, date of graduation, field of study, and university where degree was earned were extracted from publicly available faculty profiles on department websites. In our analyses, we limited our dataset to faculty members with a verified gender and date of graduation.

In China, a “faculty” refers to an academic staff that is responsible for teaching, research, and also involved in administrative duties or community service. In elite universities, a faculty member is usually someone who has a Ph.D., which is the basic qualification for academic positions in these institutions. A “tenured faculty member” is one who has successfully passed rigorous evaluations and assessments, usually after a probationary period of about six years, and has a stable position without the pressure of short-term contract renewals, often with greater academic freedom, access to better resources, and opportunities for leadership within their departments or institutions.

We focused on life sciences because women are reported to perform better in life sciences and medicine than in other fields, and the gender ratio is closest to parity in these fields [17]. This focus also facilitates comparison and analysis of our results with previous studies abroad on the gender gap in patenting within this field.

The term “Double First-Class” refers to an elite alliance of Chinese higher education institutions designated by the Ministry of Education since 2016, which aims to cultivate world-class and high-level universities as part of the “Action Plan for the Revitalization of Education in the 21st Century”. These universities are generally considered to be the elite in scientific research and higher education in China, and there are a total of 36 universities.

Based on the catalog of academic disciplines provided by the Chinese Ministry of Education, we identified departments and schools related to the life sciences. These include departments and schools of biological sciences, biotechnology, molecular biology, biochemistry, biophysics, genetics and developmental sciences, cell biology, biomedicine, botany and plant physiology, marine life, microbial sciences, molecular ecology, agricultural ecology, medical genetics, and wildlife conservation biology. We obtained the gender, title, field of research and academic background information from public university websites.

Second, we matched each faculty member’s patent information as an inventor based on their names and affiliations through the database of the Intellectual Property Search and Consultation Center of the China National Intellectual Property Administration (CNIPA). The CNIPA serves as the official intellectual property administration authority under the central government of China, and provides patent information for public and scientific purposes through its online database platform. Under Chinese intellectual property law, patents are categorized into three types: inventions, designs, and utility models.

We limited the number of patents of type “Inventions” because inventions typically require the highest level of intellectual innovation and typically take several years from filing to final grant. We used a data cutoff date of December 31, 2021, meaning that only faculty members who graduated before December 31, 2021, and whose patents were filed before that date, were included in our analyses. Unlike many previous studies, our dataset, which focuses on both the patenting and academic profiles of individual faculty in this particular field, allows us to identify and evaluate different factors that contribute to the patenting disparities between male and female faculty, thereby improving our understanding of the underlying micro-mechanisms that drive gender disparities. The final dataset contains one row per faculty with a sample size of 1836.

We created the variable “Was the graduation prior to 2008?” to identify the era in which faculty members began their academic careers, based on the timing of their highest academic degree earned. This variable serves to measure the impact, if any, of the tenure-track personnel reforms initiated in China’s top universities on patenting-related gender disparities in the country. This period marks the beginning of a substantial reform of personnel management policies in China’s elite institutions, which has dramatically increased academic productivity, as described in [24]. The variable “duration from graduation to data cut” serves as a measure of their professional lifespan in academia. The variable “number of patent applications as first inventor” is designed to assess the extent to which faculties contribute to patent applications. Being listed first, especially as the first inventor, is considered an indication of a more substantial role in the invention process of the patent, and is accordingly highly valued in performance evaluations at Chinese universities. We used “whether the faculty was involved in a patent application” as an indicator to measure the level of faculty patent activity.

The descriptive statistics of the data are presented in Table 1. The majority of faculty (68% of 1836) in the data are male. The median time from graduation to the data cut is 13.2 years. More than half of the faculty (58.3% of 1836) have filed at least one patent after graduation, and 42.7% of the faculty have at least one patent granted. In addition, the distributions for both the number of patents filed (median: 1, maximum: 599) and patents granted (median: 0, maximum: 56) are highly skewed to the right, indicating that some faculties are particularly prolific in patenting, while nearly half of them had minimal or no patenting activity. In general, faculties tend to cluster in terms of patenting, as the median number of patent applications where a faculty is the sole inventor is 0, while the median number of patent applications with multiple inventors is 5. Similar patterns are observed for patents granted (median of 0 for single-inventor patents vs. median of 3 for patents with multiple inventors). For faculty with patents filed or granted, on average, 39% of all faculty members have filed a patent as a first inventor, and 36% have been granted a patent as a first inventor, indicating that faculty tend to make significant contributions to patenting.

**Table 1:** Data description.

***Binary features***				
**Variable**	**n (%)**			
Gender *				
Male	1248 (68.0)			
Female	588 (32.0)			
Was the graduation prior to 2008? *				
Yes	929 (50.6)			
No	907 (49.4)			
Did the faculty file a patent application post-graduate? ^d^				
Yes	1078 (58.7)			
No	758 (41.3)			
Did the faculty have any patent granted post-graduate? ^d^				
Yes	760 (41.4)			
No	1076 (58.6)			
***Continous features: for all faculties***				
**Variable**	**Mean (SD)**	**Median**	**Minimum**	**Maximum**
Duration from graduation to data cut (years)	13.3 (5.28)	13.2	0.2	24.2
Number of patent applications filed from graduation to data cut ^d^	5.5 (18.02)	1	0	599
Number of patents granted from graduation to data cut ^d^	2.3 (4.98)	0	0	56
Time to first patent application post-graduation or data cut (years) ^d^	8.5 (5.43)	8	0	20
Time to first post-graduation patent grant or data cut (years) ^d^	10.6 (4.90)	10.2	0.2	20
***Continous features: for the 1078 faculties with at least 1 patent application post-graduate***				
**Variable**	**Mean (SD)**	**Median**	**Minimum**	**Maximum**
Number of patent applications with a single inventor	0.2 (1.249)	0	0	26
Proportion of patent applications with a single inventor	0.02 (0.121)	0	0	1
Number of patent applications with collabrations	9.17 (22.485)	5	0	597
Proportion of patent applications with collabrations	0.98 (0.121)	1	0	1
Number of patent applications as the first inventor ^d^	3.92 (9.019)	1	0	133
Proportion of patent applications as the first inventor ^d^	0.39 (0.381)	0.3	0	1
***Continous features: for the 760 faculties with at least 1 granted patent post-graduate***				
**Variable**	**Mean (SD)**	**Median**	**Minimum**	**Maximum**
Number of granted patents with a single inventor	0.07 (0.393)	0	0	6
Proportion of granted with a single inventor	0.02 (0.112)	0	0	1
Number of granted patents with collabrations	5.14 (6.43)	3	0	56
Proportion of granted with collabrations	0.98 (0.112)	1	0	1
Number of granted patents as the first inventor ^d^	2.17 (4.198)	1	0	39
Proportion of granted patents as the first inventor ^d^	0.36 (0.393)	0.22	0	1

*: independent variables of interest in the analyses;

^d^: variables or a combination of variables treated as dependent variables in the analyses;

variables without marks are for descriptive purposes.

**Fig 1:**
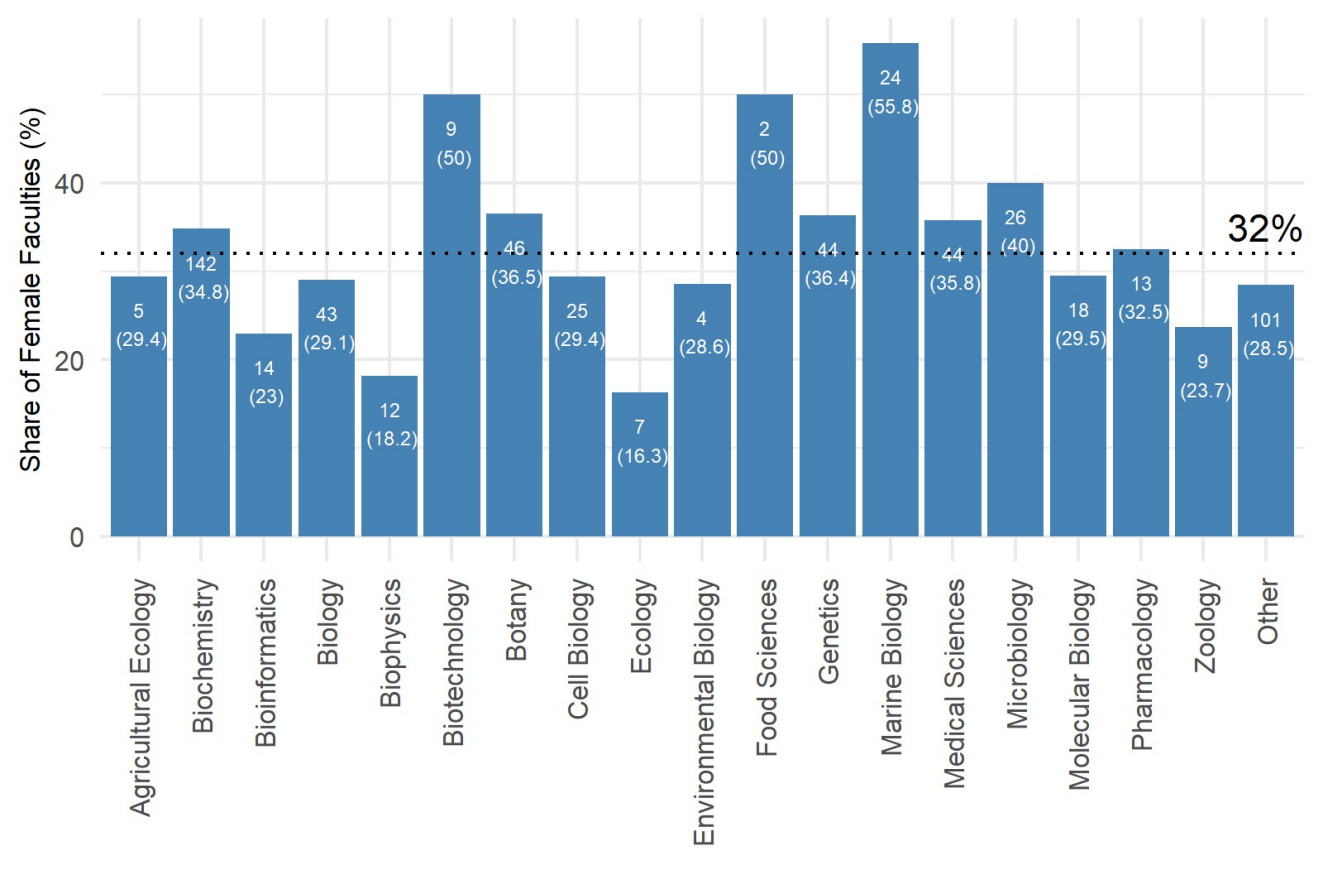
Number and percentage of female faculty members by field of research. Overall, 32% of the faculties in the data are female, and the percentage within a bar is the percentage of female faculties within the research field.

Fig 1 shows the number and percentage of female faculty by research field. Both genders are not equally represented in the different research fields. Female faculty have the highest representation in marine biology (58.2%), biotechnology (50%), and food science (50%), while they are most underrepresented in biophysics (18.2%), ecology (16.3%), and bioinformatics (23%). Women are more evenly represented in a few fields that are known to span multiple disciplines, such as pharmacology (32.5%), medical sciences (35.8%), and genetics (36.4%).

### Statistical methods

In this section, we introduce the statistical methods that are used to model the data. For the *i*^*th*^ faculty, we use the following notations in the modeling:

- *G*_*i*_: gender of the *i*^*th*^ faculty;
- *E*_*i*_: group of the *i*^*th*^ faculty, where 1 means the group of “graduation prior to 2008” and 2 means the group of “graduation on or after 2008”;
- *T*_*i*_: duration from graduation to data cut for the *i*^*th*^ faculty;
- *U*_*i*_: university of the *i*^*th*^ faculty;
- *R*_*i*_: title of the *i*^*th*^ faculty;
- *F*_*i*_: field of research for the *i*^*th*^ faculty;
- *N*_*i*_: the number of patents accumulated from graduation to data cut;
- *M*_*i*_: the number of patents that the *i*^*th*^ faculty is the first inventor.

To investigate if there is a gender gap in annual number of patents, we first estimate the overall gender gap in the annual number of patents per person after graduation using Poisson regression models, where the number of patents up to the data cutoff is the response variable and the time from graduation to the data cutoff is the independent variable, i.e,

$$\begin{array}{c} N_{i}∼\text{Poisson}\left(\lambda_{G_{i}}T_{i}\right) \end{array}$$ (1)

where λ_*M*_ and λ_*F*_ denote the annual number of patents per person for male and female faculties. Model 1 is fitted separately for both “prior to 2008” and the “on or after 2008” groups.

To perform statistical inferences on annual number of patent applications or patents granted between two genders, a separate Poisson model is used. In this model, the number of patents is the response variable; university, faculty title, and research field are the control variables; and the gender-by-group-by-time interaction is the independent variable, i.e.

$$\begin{array}{c} N_{i}∼\text{Poisson}\left(\lambda_{i}\right),\;\text{where}\;\text{log}\left(\lambda_{i}\right)=\beta_{0}+\alpha_{U_{i}}^{U}+\alpha_{R_{i}}^{R}+\alpha_{F_{i}}^{F}+\left(\beta_{G_{i}}^{G}+\beta_{E_{i}}^{E}+\gamma_{G_{i}E_{i}}\right)T_{i} \end{array}$$ (2)

where *β*_0_ is the intercept; *α*^*U*^’s, *α*^*R*^’s, and *α*^*F*^’s are the coefficients for the control variables of university, title and research field; *β*_*G*_’s and *β*^*E*^’s are the main effects for gender and group; *γ* is the effect of the gender-by-group interaction. A logarithm link function is used to ensure model convergence.

To assess whether there is any gender difference in the contributions to patenting by gender, we consider how often faculty members contribute as first inventors. We estimate the probability of being the first inventor on a patent using generalized linear models with binomial distributions, i.e,

$$\begin{array}{c} M_{i}∼\text{Binomial}\left(N_{i},p_{G_{i}}\right) \end{array}$$ (3)

where *p*_*M*_ and *p*_*F*_ denote the probability of being the first inventor for male and female faculties. The model is fitted separately for the “before 2008” and the “on or after 2008” groups. Statistical inference between the two genders is performed using another generalized linear model that includes university, faculty title, and research field as control variables, and the interaction between gender and group as the independent variable, i.e.

$$\begin{array}{c} M_{i}∼\text{Binomial}\left(N_{i},p_{i}\right),\;\text{where}\;\text{logit}\left(p_{i}\right)=\beta_{0}+\alpha_{U_{i}}^{U}+\alpha_{R_{i}}^{R}+\alpha_{F_{i}}^{F}+\beta_{G_{i}}^{G}+\beta_{E_{i}}^{E}+\gamma_{G_{i}E_{i}} \end{array}$$ (4)

where *β*_0_ is the intercept; *α*^*U*^’s, *α*^*R*^’s, and *α*^*F*^’s are the coefficients for the university, title, and research field control variables; *β*_*G*_’s and *β*^*E*^’s are the main effects of gender and group; *γ* is the effect of the gender-by-group interaction. A logit link function is used to ensure model convergence.

We further segment the patents by the time of application after graduation into the categories “0–5 years”, “6–10 years”, and “11–15 years” to examine any change in the gender gap in patenting behavior with career progression. The annual number of patents per person after graduation is analyzed similarly to model 2. The contributions to patenting are then analyzed using generalized linear models as in model 4.

Regarding the gender differences in the timing of patenting, we also treat the timing of the first patent after graduation as a time-to-event endpoint. Kaplan-Meier curves are plotted for each gender, with the goal of estimating the probability that a faculty member has not filed a patent (or had a patent granted) by a certain time after graduation. The time to first patent application (or first patent grant) is fitted in a Cox proportional hazards model that includes university, title, research field, gender, group, and gender by group interaction, i.e,

$$\begin{array}{c} h_{i}\left(t\right)=h_{0}\left(t\right)\;\text{exp}\left\{\alpha_{U_{i}}^{U}+\alpha_{R_{i}}^{R}+\alpha_{F_{i}}^{F}+\beta_{G_{i}}^{G}+\beta_{E_{i}}^{E}+\gamma_{G_{i}E_{i}}.\right\} \end{array}$$ (5)

If the interaction between gender and group is not statistically significant, it is removed and hazard ratios between genders and between the “before 2008” and “on or after 2008” groups are derived using the updated model.

In our analyses, the statistical significance is claimed if the corresponding p-value is less than 0.05.

## Results

### Gender gap in annual number of patents per person

**Fig 2:**
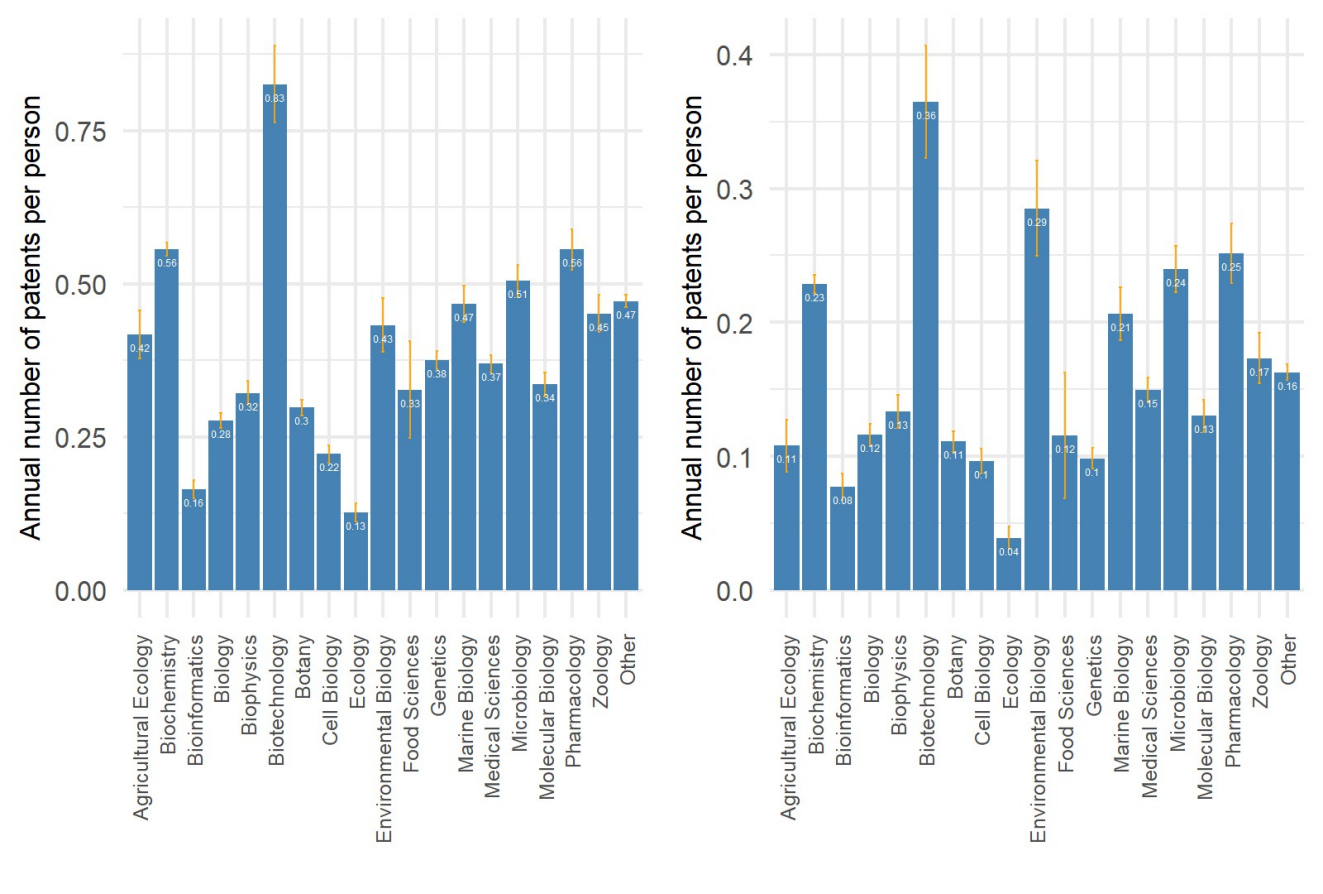
Estimated annual number of patent applications and patents granted per person by field of research. Left: annual number of patent applications per person; right: annual number of patents granted per person. The annual number of patents is estimated by Poisson regression, where the number of patents is the response variable and the field of research and the time since graduation are the independent variables.

Fig 2 shows the estimated annual number of patent applications and patents granted per person by field of research. It is clear that patenting activity is not uniform across fields. For example, biotechnology has the highest annual number of patent applications and patents granted, while ecology has the lowest. This partly justifies controlling for field of research when making statistical inferences about patenting.

**Fig 3:**
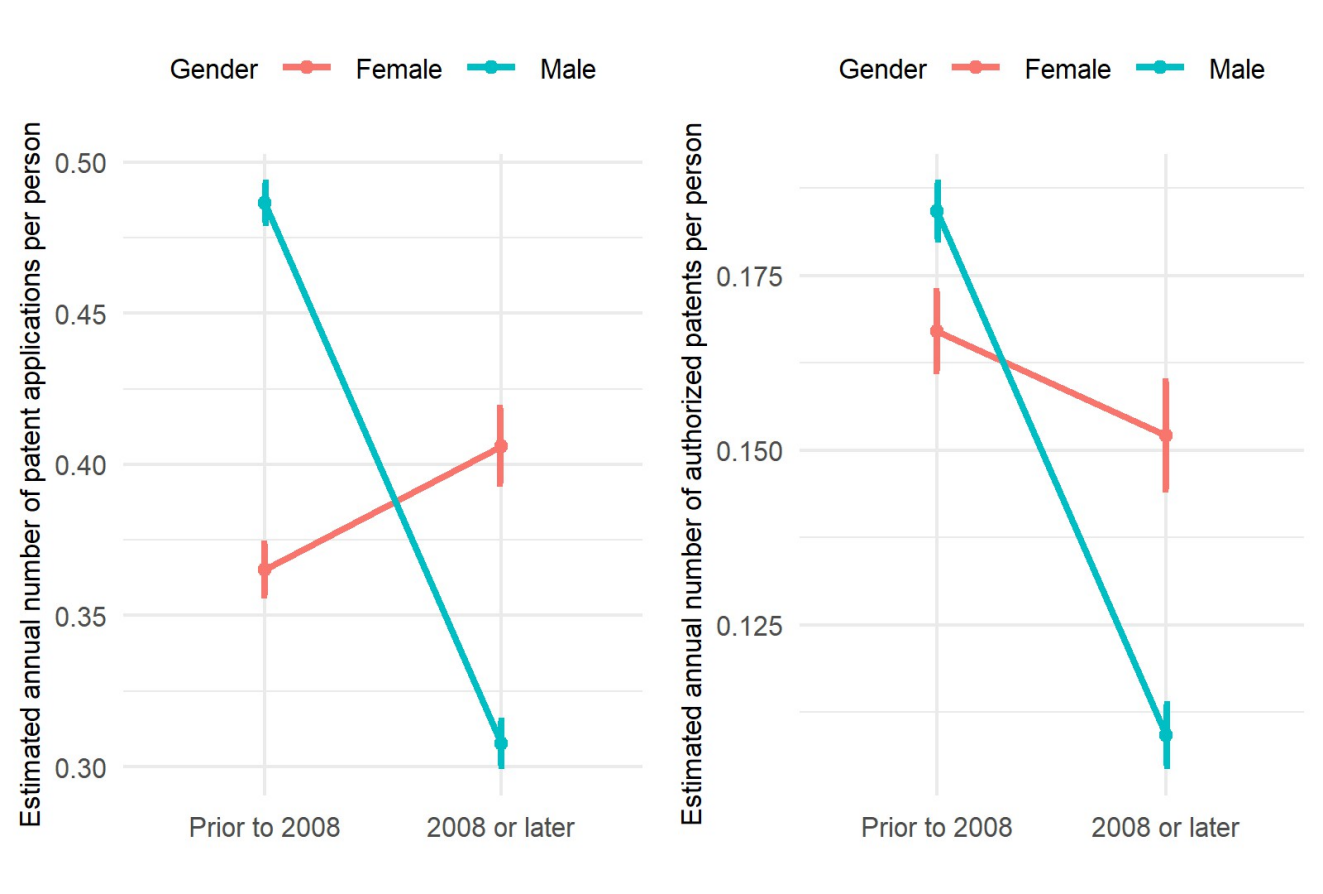
Estimated annual number of patent applications and patents granted per person by gender and time of graduation. Left: annual number of patent applications per person; right: annual number of patents granted per person. The annual number of patents is estimated by Poisson regression, where the number of patents is the response variable and the interaction of gender by time of study is the independent variable.

The number of patents filed per year is estimated using Poisson regression (Fig 3). For faculties graduated before 2008, men had a significantly higher number of patents filed per year after graduation than women (men: 0.49, women: 0.36, p-value: <0.001). This pattern is reversed for faculties graduated in 2008 or later, where women outnumbered men in patents filed per year (men: 0.31, women: 040, p-value: <0.001). Comparing the gender gaps between the “before 2008” and “on or after 2008” groups also shows that the change in gender gaps is statistically significant (p-value <0.001). For granted patents, male faculties also had a slightly higher annual number of patents in the “before 2008” group (male: 0.18, female: 0.17, p-value: 0.0764) and female faculties outperformed male faculties in the “on or after 2008” group (male: 0.11, female: 0.15, p-value: <0.001). The details of the Poisson regression results for statistical inference are listed in Tables 2 and 3.

**Table 2:** Poisson regression results on the annual number of patent applications per person.

Parameter	Estimate	S. E.	p-value
(Intercept)	-0.37	0.114	0.001
Title—Associate Professor	0.21	0.05	<0.001
Title—Professor	0.59	0.05	<0.001
Title—Unknown	0.88	0.176	<0.001
Field—Agricultural Ecology	-0.43	0.113	<0.001
Field—Biochemistry	-0.14	0.063	0.023
Field—Bioinformatics	-1.3	0.108	<0.001
Field—Biology	-0.83	0.076	<0.001
Field—Biophysics	-0.68	0.087	<0.001
Field—Biotechnology	0.16	0.104	0.128
Field—Botany	-0.58	0.076	<0.001
Field—Cell Biology	-0.64	0.089	<0.001
Field—Ecology	-1.77	0.134	<0.001
Field—Environmental Biology	-0.39	0.12	0.001
Field—Food Sciences	-0.57	0.252	0.023
Field—Genetics	-0.54	0.073	<0.001
Field—Marine Biology	-0.8	0.107	<0.001
Field—Medical Sciences	-0.49	0.073	<0.001
Field—Microbiology	-0.15	0.079	0.063
Field—Molecular Biology	-0.52	0.085	<0.001
Field—Other	-0.11	0.066	0.092
Field—Zoology	-0.27	0.092	0.003
Group—1: duration	0.08	0.003	<0.001
Group—2: duration	0.07	0.006	<0.001
Gender—Female: duration	-0.02	0.002	<0.001
Group—2: Gender—Female: duration	0.03	0.004	<0.001

In the model, the number of patent applications is the response variable; university, faculty title, and research field are the control variables; the gender-by-group-by-time interaction is the independent variable. Etimates for university-related control variables are not shown due to the large number of variables and the control nature of the variables.

**Table 3:** Poisson regression results of annual number of granted patents per person.

Parameter	Estimate	S. E.	p-value
Intercept	-1.27	0.171	<0.001
Title—Associate Professor	0.52	0.085	<0.001
Title—Professor	0.7	0.086	<0.001
Title—Unknown	0.96	0.277	<0.001
Field—Agricultural Ecology	-0.89	0.207	<0.001
Field—Biochemistry	-0.23	0.095	0.014
Field—Bioinformatics	-1.19	0.159	<0.001
Field—Biology	-0.8	0.116	<0.001
Field—Biophysics	-0.58	0.132	<0.001
Field—Biotechnology	0.04	0.157	0.778
Field—Botany	-0.77	0.118	<0.001
Field—Cell Biology	-0.64	0.134	<0.001
Field—Ecology	-1.89	0.233	<0.001
Field—Environmental Biology	0.03	0.157	0.866
Field—Food Sciences	-0.91	0.421	0.031
Field—Genetics	-0.99	0.119	<0.001
Field—Marine Biology	-1.04	0.158	<0.001
Field—Medical Sciences	-0.56	0.111	<0.001
Field—Microbiology	-0.17	0.117	0.149
Field—Molecular Biology	-0.67	0.131	<0.001
Field—Other	-0.32	0.1	0.001
Field—Zoology	-0.46	0.143	0.001
Group—1: duration	0.1	0.005	<0.001
Group—2: duration	0.09	0.009	<0.001
Gender—Female: duration	0	0.002	0.076
Group—2: Gender—Female: duration	0.03	0.007	<0.001

In the model, the number of patents granted is the response variable; university, faculty title, and research field are the control variables; and the gender-by-group-by-time interaction is the independent variable. Estimates for the university-related control variables are not shown due to the large number of variables and the control nature of the variables.

**Fig 4:**
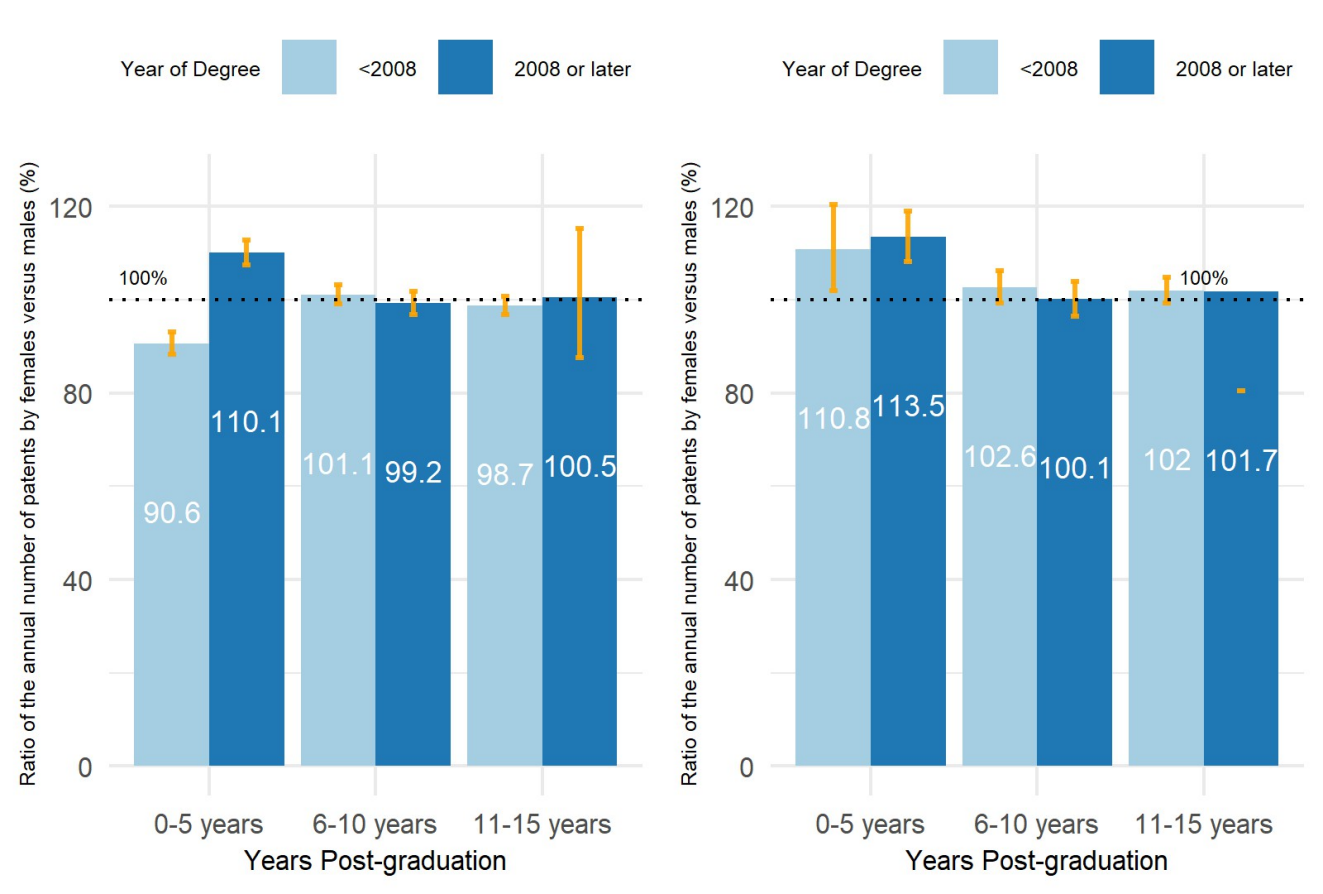
Estimated ratio of annual number of patent applications and granted patents per person between male and female faculties with 95% CIs. Left: patent applications; right: patents granted. The ratios are estimated using generalized linear models with Poisson distributions. The number of patent applications within the given time period is the response variable, and university, title, field, and gender within the given time period are independent variables.

We segment the number of patent applications by time period after graduation to further examine when gender gaps occurred. As shown in Fig 4, for patent applications, the overall pattern of gender gaps was mainly driven by the early stage of the career (0–5 years after graduation). No significant differences were found for the period 5–10 years after graduation, and male faculty have more patent applications in the long term (10–15 years after graduation). For granted patents, female faculty actually had significantly more granted patents at the early stage of their careers (0–5 years after graduation), and there was no significant difference between the two genders in the long term.

### Contribution-specific gender differences

**Fig 5:**
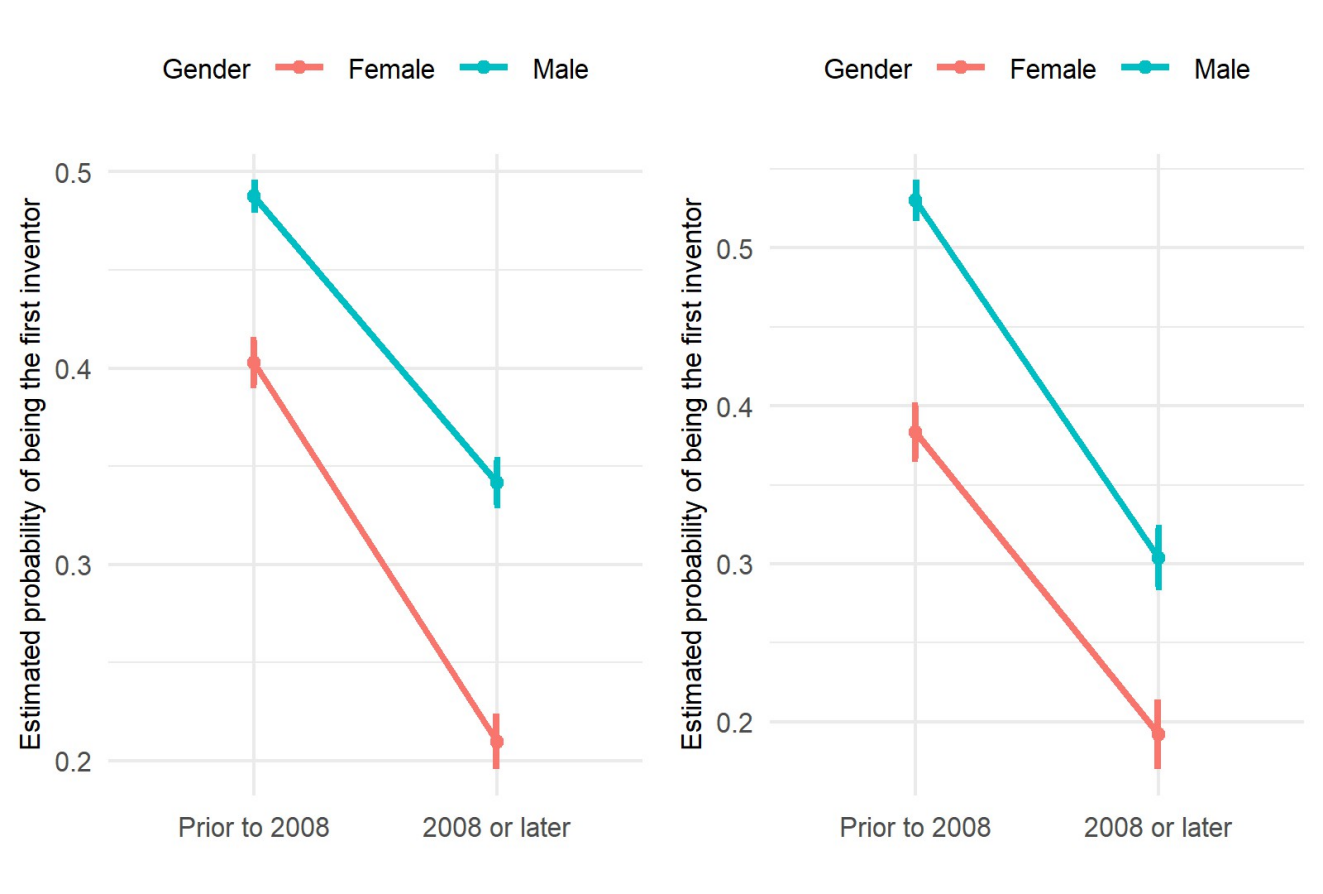
Estimated probability of a faculty being the first inventor of a patent. Left: patent applications; right: granted patents. The probabilities are estimated using generalized linear models with binomial distributions. The number of patents and the number of patents where the faculty is the first inventor together are the response variables, and group, gender, and the interaction between group and gender are the independent variables.

Fig 5 shows the likelihood of a faculty member being the first inventor on a patent. For both the “before 2008” group (male: 49%, female: 40%, p-value: <0.001) and the “on or after 2008” group (male: 34%, female: 21%, p-value: <0.001), male faculty are significantly more likely to be the first inventor on a patent application. A similar pattern was observed for granted patents, where male faculty had significant odds of being the first inventor in both the “before 2008” group (male: 53%, female: 38.3%, p-value: <0.0001) and “on or after 2008” group (male: 30.4%, female: 19.2%, p-value: <0.001). Details of the statistical inferences are given in Tables 4 and 5.

**Table 4:** Generalized regression results of probability of being the first inventor in a patent application.

Parameter	Estimate	S. E.	p-value
Intercept	-0.92	0.236	<0.001
Title—Associate Professor	0.57	0.127	<0.001
Title—Professor	1	0.125	<0.001
Title—Unknown	1.2	0.372	0.001
Field—Agricultural Ecology	0.95	0.265	<0.001
Field—Biochemistry	0.05	0.136	0.696
Field—Bioinformatics	-0.3	0.233	0.196
Field—Biology	-0.19	0.165	0.245
Field—Biophysics	0.7	0.193	<0.001
Field—Biotechnology	0.1	0.23	0.661
Field—Botany	0.3	0.17	0.077
Field—Cell Biology	-0.45	0.205	0.029
Field—Ecology	0.8	0.291	0.006
Field—Environmental Biology	-0.37	0.253	0.143
Field—Food Sciences	-0.26	0.511	0.608
Field—Genetics	-0.27	0.159	0.093
Field—Marine Biology	-0.99	0.247	<0.001
Field—Medical Sciences	-0.02	0.157	0.917
Field—Microbiology	-0.31	0.17	0.068
Field—Molecular Biology	-0.01	0.182	0.974
Field—Other	-0.53	0.141	<0.001
Field—Zoology	0.64	0.207	0.002
Group—2	-0.52	0.069	<0.001
Gender—Female	-0.23	0.063	<0.001
Group—2: Gender—Female	-0.34	0.114	0.003

In the model, the number of patent applications and the number of applications where the faculty is an inventor together are the response variables; university, faculty title, and research field are the control variables; gender, group, and the interaction of gender and group are the independent variables. Binomial distributions with a logit link function are used. Estimates for university-related control variables are not shown due to the large number of variables and the control nature of the variables.

**Table 5:** Generalized regression results of probability of being the first inventor in a granted application.

Parameter	Estimate	S. E.	p-value
Intercept	-0.78	0.35	0.026
Title—Associate Professor	0.66	0.23	0.004
Title—Professor	1.26	0.228	<0.001
Title—Unknown	1.93	0.611	0.002
Field—Agricultural Ecology	-0.15	0.446	0.744
Field—Biochemistry	-0.41	0.204	0.043
Field—Bioinformatics	-0.7	0.349	0.046
Field—Biology	-0.53	0.253	0.035
Field—Biophysics	-0.23	0.293	0.439
Field—Biotechnology	-0.21	0.342	0.535
Field—Botany	-0.04	0.257	0.873
Field—Cell Biology	-0.78	0.312	0.013
Field—Ecology	0.28	0.538	0.607
Field—Environmental Biology	-0.53	0.35	0.129
Field—Food Sciences	-0.45	0.869	0.605
Field—Genetics	-1.02	0.265	<0.001
Field—Marine Biology	-1.14	0.357	0.001
Field—Medical Sciences	-0.54	0.241	0.025
Field—Microbiology	-0.59	0.256	0.022
Field—Molecular Biology	-0.37	0.285	0.191
Field—Other	-0.38	0.209	0.066
Field—Zoology	0.32	0.319	0.323
Group—2	-0.74	0.117	<0.001
Gender—Female	-0.43	0.095	<0.001
Group—2: Gender—Female	-0.02	0.19	0.929

In the model, the number of patents granted and the number of patents granted where the faculty is an inventor together are the response variables; university, faculty title, and research field are the control variables; gender, group, and the interaction of gender and group are the independent variables. Binomial distributions with a logit link function are used. Estimates for university-related control variables are not shown due to the large number of variables and the control nature of the variables.

**Fig 6:**
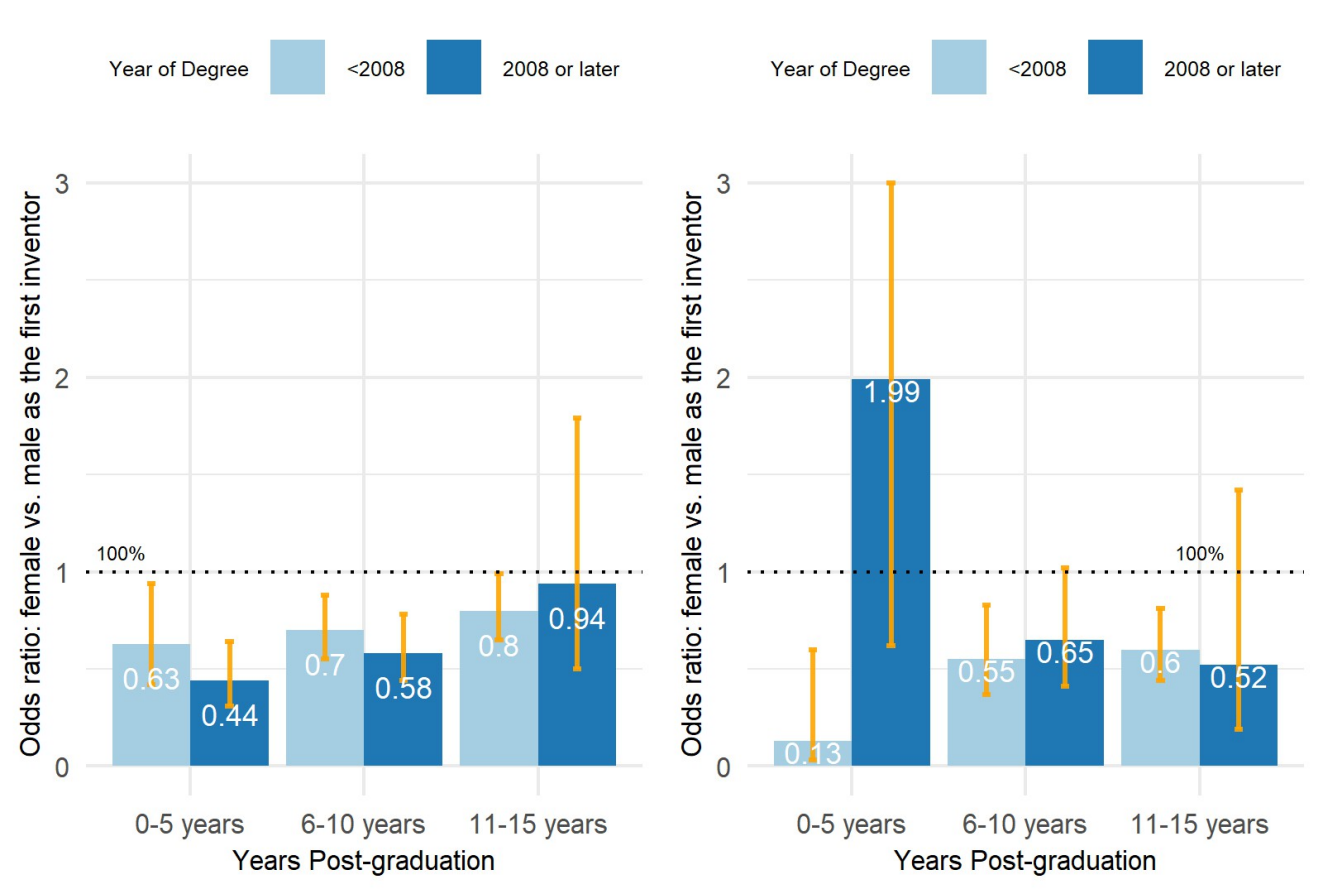
Estimated odds ratio of being the first inventor between female and male faculties with 95% CIs. Left: patent applications; Right: granted patents. Odds ratios are estimated using generalized linear models with binomial distributions. The number of patents and the number of patents in which the faculty member is the first inventor combined are the response variables. University, title, field, gender, group, and the interaction of gender and group are the independent variables.

This suggests that male faculties tend to be in a more dominant position in contributing to patenting. Fig 6 shows the odds ratio of being the first inventor between female and male faculties. For patent applications, female faculty had a lower probability of being the first inventor compared to their male counterparts for all time periods, and this probability increased with career progression. Similar patterns were observed for patents granted, except that for faculty with degrees awarded in 2008 or later, female faculty were more likely to be first inventors in the early career stage, although the advantage was not statistically significant.

### Gender differences in time to first patent after graduation

**Fig 7:**
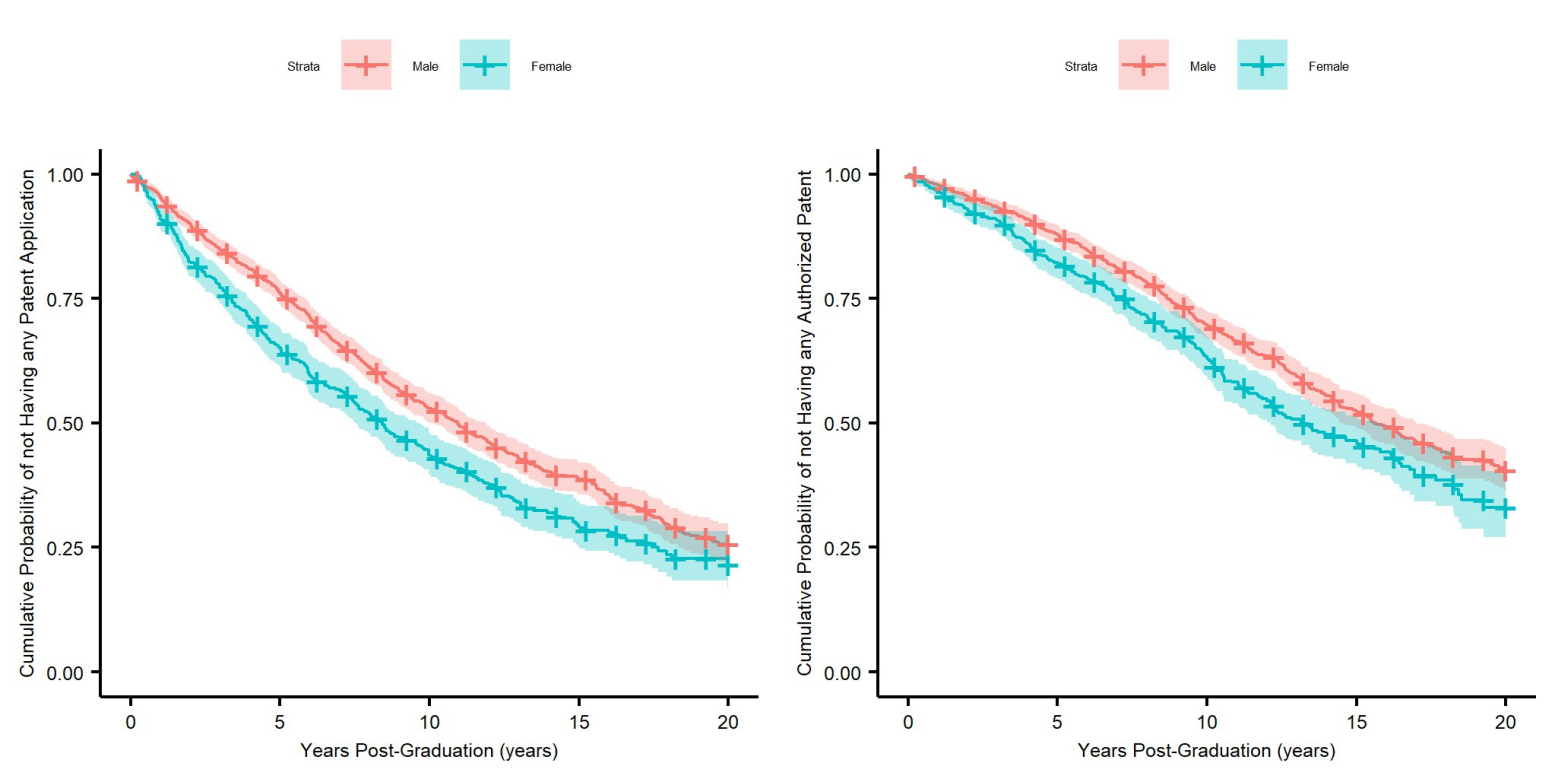
Kaplan-Meier estimates of time to first patent after graduation by gender. Left: Time to first patent application; Right: Time to first patent grant.

Fig 7 plots the probability that a faculty has not filed a patent application by time using Kaplan-Meier estimates. At all time points after graduation, male faculty have a higher cumulative probability of having not patented yet, suggesting that female faculty engage in patenting activities significantly earlier. The Cox proportional hazards models with control variables of university, title and research field, as well as the main effects of gender, group, and gender-by-group interaction yielded a non-significant gender-by-group interaction effect (p-value for patent applications: 0.68; p-value for patents granted: 0.59), so the interaction term is removed from the models. From Tables 6 and 7, it can also be seen that male faculty have a significantly higher probability of not filing a patent application after graduation than female faculty (HR (95% C.I.: 1.21 (1.06, 1.38), p-value: 0.005). Furthermore, faculties that graduated on or after 2008 took significantly less time to file the first postgraduate patent application (HR (95% C.I.): 1.21 (1.05, 1.40), p-value: 0.009). Similar patterns are identified for first granted patents.

**Table 6:** Cox proportional hazards regression results of time to first patent application.

Parameter	Estimate	S. E.	p-value
Title—Associate Professor	0.18	0.124	0.149
Title—Professor	0.03	0.125	0.819
Title—Unknown	0.83	0.499	0.098
Field—Agricultural Ecology	-0.29	0.338	0.399
Field—Biochemistry	-0.32	0.19	0.096
Field—Bioinformatics	-0.88	0.259	<0.001
Field—Biology	-0.79	0.218	<0.001
Field—Biophysics	-1.09	0.267	<0.001
Field—Biotechnology	0.03	0.349	0.926
Field—Botany	-0.62	0.225	0.006
Field—Cell Biology	-0.83	0.242	<0.001
Field—Ecology	-1.6	0.347	<0.001
Field—Environmental Biology	-0.3	0.369	0.422
Field—Food Sciences	-0.51	0.74	0.487
Field—Genetics	-0.76	0.221	<0.001
Field—Marine Biology	-0.84	0.315	0.008
Field—Medical Sciences	-0.8	0.222	<0.001
Field—Microbiology	-0.15	0.235	0.515
Field—Molecular Biology	-0.42	0.241	0.078
Field—Other	-0.54	0.2	0.007
Field—Zoology	-0.94	0.307	0.002
Gender—Female	0.18	0.068	0.007
Group—2	0.23	0.073	0.002

In the model, time from graduation to first post-graduation patent application are the response variables; university, faculty title, and research field are the control variables; and gender and group are the independent variables. The interaction between gender and group is not included because it is not statistically significant in the full model, and the estimates for the university-related control variables are not shown because of the large number of variables and the control nature of the variables.

**Table 7:** Cox proportional hazards regression results of time to first patent application.

Parameter	Estimate	S. E.	p-value
Title—Associate Professor	0.29	0.162	0.072
Title—Professor	0.06	0.164	0.724
Title—Unknown	0.86	0.642	0.181
Field—Agricultural Ecology	-0.4	0.406	0.326
Field—Biochemistry	-0.26	0.228	0.248
Field—Bioinformatics	-0.76	0.315	0.016
Field—Biology	-0.79	0.268	0.003
Field—Biophysics	-1.15	0.348	<0.001
Field—Biotechnology	0.46	0.391	0.244
Field—Botany	-0.42	0.266	0.112
Field—Cell Biology	-0.71	0.297	0.017
Field—Ecology	-1.76	0.469	<0.001
Field—Environmental Biology	-0.25	0.441	0.574
Field—Food Sciences	0.65	0.753	0.387
Field—Genetics	-0.82	0.269	0.002
Field—Marine Biology	-0.58	0.364	0.109
Field—Medical Sciences	-0.66	0.267	0.014
Field—Microbiology	0.03	0.273	0.918
Field—Molecular Biology	-0.45	0.29	0.118
Field—Other	-0.39	0.241	0.102
Field—Zoology	-0.92	0.376	0.014
Gender—Female	0.18	0.08	0.024
Group—2	0.35	0.09	<0.001

In the model, time from graduation to first post-graduation patent granted are the response variables; university, faculty title, and research field are the control variables; gender and group are the independent variables. The interaction between gender and group is not included because it is not statistically significant in the full model. Estimates for the university-related control variables are not shown due to the large number of variables and the control nature of the variables.

## Discussion

Our study reveals the evolving patenting patterns of academic researchers in the life sciences at China’s top universities. On the one hand, the gender gap at the individual level, in terms of the annual number of patent applications and patents granted per person, shows a trend of gradual narrowing. Specifically, among researchers who joined the university before the tenure-track reform, men significantly outnumbered women in terms of the annual number of patents per person. However, this trend has shifted in the post-reform period; female researchers have started to file a significantly higher number of patents per year, with this increase being particularly pronounced in the first five years after graduation. In addition, female faculty members show a propensity for earlier involvement in the patenting process than their male counterparts, a discrepancy that remains consistent over time. One possible explanation for this trend could be that female faculty members may perceive early patenting as a strategic move to enhance their academic portfolio. This contrasts sharply with the patterns identified by [9] for American scientists. One explanation for this discrepancy could be cultural differences in the academic environments of China and the United States, which may influence gender dynamics and the accessibility of resources for patenting.

On the other hand, this study highlights persistent gender disparities in patent inventor rankings, which serve as indicators of contribution and status within research teams. Despite progress, women remain less likely to achieve prominent roles such as principal investigator or team leader both before and after 2008, a gap that does not significantly close even as female faculty gain experience.

These findngs reveals significant progress in traditionally male-dominated STEM fields, and highlights the extensive efforts needed to overcome gender barriers. The narrowing gender gap in academic patenting reflects a broader trend in China, where women have gained greater access to higher education and academic careers. From the late 1990s to the early 2000s, the proportion of female students in higher education increased significantly from 39.66% to 49.86% [58]. Today, women account for 50% of all undergraduate students and 51.2% of graduate students [59], 56.57% of students studying abroad [60], and 49.59% of full-time faculty members at universities [61].

Compared to other STEM fields in China and elsewhere, the life sciences sector has a higher representation of women, more opportunities, and less pronounced gender disparities [17]. The life sciences have become a favorable arena for women’s empowerment and recognition in China, as evidenced by the increasing presence of female scientists and academics in social media and public discussions [62]. This visibility not only highlights their achievements, but also supports a narrative that emphasizes women’s capabilities and the need for gender equality. Overall, the life sciences are showing signs of moving away from traditional gender biases, with the gender gap not only narrowing but potentially reversing, heralding significant shifts in gender dynamics within science and technology.

The tenure-track system reform in China’s elite universities can be seen as a significant step toward bridging the gender gap in academia. Under its merit-based evaluation criteria, patents are a valued component of academic evaluation. These changes for qualifying tenure-track faculty members allow female researchers, who may have faced more subjective barriers to advancement in the past, to compete with their male counterparts on a more level playing field. This reform may also encourage female faculty to become more active in research and patenting, an area where women can unleash latent innovative potential and make their contributions more visible, as successful cases of women thriving under these rigorous conditions.

Despite these positive changes, our research also reveals a deep-seated gender gap in the depth of contributions and the nature of involvement in patenting activities. Men tend to start patenting later in their careers, but are more likely to move into leadership roles within patenting teams. Conversely, women often start patenting earlier but are often assigned to support roles. This pattern is consistent with previous studies suggesting that increased representation of women in higher education does not translate into their achieve proportional representation at higher levels. The “leaky pipeline” phenomenon is particularly severe in China, where the proportion of women decreases at each academic stage from graduate student to full-time faculty [17], and women’s representation is particularly pronounced at the associate and full professor levels significant decline [63]. This may be related to a gendered hierarchy within the collaborative ventures of the academic industry, which continues to hinder women’s chances of securing senior positions. However, this disadvantage may also be related to the challenges women faculty face in balancing family responsibilities and academic commitments early in their careers. As they progress in academia, their dominance in patenting gradually increases, reflecting previous research showing that women tend to achieve senior titles and elite researcher status later in their careers compared to their male counterparts [17].

## Conclusion

Our study highlights a narrowing gender gap in patenting activity among life science faculty members from top Chinese universities. This trend correlates with tenure-track reforms in Chinese higher education and may also be related to increased opportunities for women to participate in the life sciences and a more female-friendly work environment. However, the increasing prominence of female faculty members has not yet significantly changed the existing gender structure within research and patenting collaborations.

Our study is limited by its narrow focus on elite Chinese universities and the life sciences sector, which may not reflect the dynamics present in other disciplines or different types of academic institutions. In order to increase the generalizability of our findings, future research should expand to different disciplines and include a more diverse range of universities. In addition, we found that patenting behavior varies significantly across life science disciplines, suggesting the influence of different disciplinary cultures, resource allocation, and institutional support. Recognizing these subtleties is essential for designing targeted policies and interventions that promote equitable innovation across the scientific community.

The study highlights the need for policies in China aimed at increasing women’s leadership in academic invention, which is essential for creating a gender-inclusive and balanced research environment. It recommends that Chinese academic institutions initiate targeted mentorship programs that pair female faculty with experienced senior inventors, focusing on developing leadership skills, navigating the patent application process, and fostering research collaborations. In addition, the Department of Education should establish incentive structures specifically designed to reward female faculty for lead inventorship. These incentives could include grants, research funding, and recognition awards, all aimed at encouraging and honoring women who take a lead role in patent applications. These initiatives are critical to promoting gender equity in academia and science in China.

In light of previous research indicating a relationship between increased educational opportunities for women and fertility decline [64, 65]. It is quite likely that female faculty must prioritize their education and career advancement over marriage and childbearing in the early stage of their careers, and coupled with societal expectations for women to balance work and family [66], this may lead to lower fertility rates among highly educated women [67]. While our study lacks the data to explore this issue further, these issues deserve considerable attention in future studies, along with considerations of appropriate support structures for early-career women faculty.

## Supporting information

S1 Data (DOCX)

## References

[1] Ross MB, Glennon BM, Murciano-Goroff R, Berkes EG, Weinberg BA, Lane JI. Women are credited less in science than men. Nature. 2022;608:135–145. doi: 10.1038/s41586-022-04966-w 35732238 PMC9352587

[2] Chavatzia T. Cracking the code: Girls’ and women’s education in science, technology, engineering and mathematics (STEM). Paris, France: United Nations Educational, Scientific and Cultural Organization. 2017;.

[3] Bentley JT, Adamson R. Gender Differences in the Careers of Academic Scientists and Engineers: A Literature Review. Special Report.; 2003.

[4] Cole NS. Bias in Selection. Journal of Educational Measurement. 1973;10(4):237–255. doi: 10.1111/j.1745-3984.1973.tb00802.x

[5] Link AN, Siegel DS, Bozeman B. An empirical analysis of the propensity of academics to engage in informal university technology transfer*. Industrial and Corporate Change. 2007;16(4):641–655. doi: 10.1093/icc/dtm020

[6] Sonnert G, Holton G. Who Succeeds in Science?: The Gender Dimension. Rutgers University Press; 1995.

[7] Xie Y, Shauman KA. Sex differences in research productivity: New evidence about an old puzzle. American sociological review. 1998; p. 847–870. doi: 10.2307/2657505

[8] Organization WIP, Alemán M. The Global Gender Gap in Innovation and Creativity: An International Comparison of the Gender Gap in Global Patenting over Two Decades. Geneva, Switzerland: World Intellectual Property Organization; 2023.

[9] Ding WW, Murray F, Stuart TE. Gender differences in patenting in the academic life sciences. science. 2006;313(5787):665–667. doi: 10.1126/science.1124832 16888138

[10] Frietsch R, Haller I, Funken-Vrohlings M, Grupp H. Gender-specific patterns in patenting and publishing. Research Policy. 2009;38(4):590–599. doi: 10.1016/j.respol.2009.01.019

[11] Mauleón E, Daraio C, Bordons M. Exploring gender differences in patenting in Spain. Research Evaluation. 2013;23(1):62–78. doi: 10.1093/reseval/rvt030

[12] Bunker Whittington K, Smith-Doerr L. Women Inventors in Context: Disparities in Patenting Across Academia and Industry. Gender and Society—GENDER SOC. 2008;22:194–218. doi: 10.1177/0891243207313928

[13] Hunt J, Garant JP, Herman H, Munroe DJ. Why Don’t Women Patent? National Bureau of Economic Research; 2012. 17888. Available from: http://www.nber.org/papers/w17888.

[14] Ceci SJ, Williams WM. Understanding current causes of women’s underrepresentation in science. Proceedings of the National academy of sciences. 2011;108(8):3157–3162. doi: 10.1073/pnas.1014871108 21300892 PMC3044353

[15] Whittington KB. Mothers of invention? Gender, motherhood, and new dimensions of productivity in the science profession. Work and Occupations. 2011;38(3):417–456. doi: 10.1177/0730888411414529

[16] Notice on Several Measures to Support Female Scientific and Technological Talents in Playing a Greater Role in Technological Innovation. Ministry of Science and Technology; 2021.

[17] Gender in the China Research Arena. National Science Library, Chinese Academy of Sciences, & Elsevier; 2022.

[18] Yun Zhao DL, Zhou Y. The performance of Chinese female scholars: A gendered study on the publication rate in journalism and communication field from 2011 to 2020. Cogent Social Sciences. 2023;9(1):2163966. doi: 10.1080/23311886.2022.2163966

[19] Tao Y, Hong W, Ma Y. Gender differences in publication productivity among academic scientists and engineers in the US and China: similarities and differences. Minerva. 2017;55:459–484. doi: 10.1007/s11024-017-9320-6

[20] Rinaldo N, Piva G, Ryder S, Crepaldi A, Pasini A, Caruso L, et al. The Issue of Gender Bias Represented in Authorship in the Fields of Exercise and Rehabilitation: A 5-Year Research in Indexed Journals. Journal of Functional Morphology and Kinesiology. 2023;8. doi: 10.3390/jfmk8010018 36810502 PMC9944464

[21] Xie Z, Zhang X. The patterns of patents in China. China Economic Journal. 2015;8(2):122–142. doi: 10.1080/17538963.2015.1046219

[22] Zhang Z, Zong Q. Gender diversity and patent quality: Evidence from Chinese patent data. International Studies of Economics. 2023;18(4):430–453. doi: 10.1002/ise3.54

[23] Kou M, Zhang Y, Zhang Y, Chen K, Guan J, Xia S. Does gender structure influence R&D efficiency? A regional perspective. Scientometrics. 2020;122:477–501. doi: 10.1007/s11192-019-03282-x

[24] Zhang J. Developing excellence: Chinese university reform in three steps. Nature. 2014;514(7522):295–296. doi: 10.1038/514295a 25318507

[25] From 2012 to 2021, the number of patent applications in universities increased to 367,000, an increase of 246.2 Available from: http://news.china.com.cn/2022-08/25/content_78389128.html.

[26] China Patent Survey Report. Intellectual property development & research center, China national intellectual property administration; 2022.

[27] Delgado M, Mariani M, Murray F. The Role of Location on the Inventor Gender Gap: Women are Geographically Constrained; 2019. Available from: https://conference.druid.dk/Druid/?confId=59.

[28] Miguelez E, Toole A, MYERS A, Breschi S, Ferruci E, Lissoni F, et al. Progress and Potential: A profile of women inventors on U.S. patents. U.S. Patent and Trademark Office; 2019.

[29] Women’s participation in inventive activity Evidence from EPO data. European Patent Office; 2022.

[30] Wang Y, Yang Z, Liu L, Wang X. Gender bias in patenting process. Journal of Informetrics. 2020;14(3):101046. doi: 10.1016/j.joi.2020.101046

[31] Milli J, Gault B, Williams-Baron E, Xia J, Berlan M. The gender patenting gap. Institute for Women’s Policy Research. 2016;.

[32] Jensen K, Kovács B, Sorenson O. Gender differences in obtaining and maintaining patent rights. Nature biotechnology. 2018;36(4):307–309. doi: 10.1038/nbt.4120 29621210

[33] Filardo G, Da Graca B, Sass DM, Pollock BD, Smith EB, Martinez MAM. Trends and comparison of female first authorship in high impact medical journals: observational study (1994-2014). bmj. 2016;352. 26935100 10.1136/bmj.i847 PMC4775869

[34] Lerchenmüller C, Lerchenmueller MJ, Sorenson O. Long-term analysis of sex differences in prestigious authorships in cardiovascular research supported by the National Institutes of Health. Circulation. 2018;137(8):880–882. doi: 10.1161/CIRCULATIONAHA.117.032325 29459476

[35] Whittington KB, Smith-Doerr L. Gender and commercial science: Women’s patenting in the life sciences. The Journal of Technology Transfer. 2005;30(4):355–370. doi: 10.1007/s10961-005-2581-5

[36] Hunt J, Garant JP, Herman H, Munroe DJ. Why are women underrepresented amongst patentees? Research Policy. 2013;42(4):831–843. doi: 10.1016/j.respol.2012.11.004

[37] Lane KA, Goh JX, Driver-Linn E. Implicit science stereotypes mediate the relationship between gender and academic participation. Sex Roles. 2012;66:220–234. doi: 10.1007/s11199-011-0036-z

[38] Fox MF. Gender, family characteristics, and publication productivity among scientists. Social studies of science. 2005;35(1):131–150. doi: 10.1177/0306312705046630

[39] Goulden M, Mason MA, Frasch K. Keeping women in the science pipeline. The ANNALS of the American Academy of Political and Social Science. 2011;638(1):141–162. doi: 10.1177/0002716211416925

[40] Cech EA, Blair-Loy M. The changing career trajectories of new parents in STEM. Proceedings of the National Academy of Sciences. 2019;116(10):4182–4187. doi: 10.1073/pnas.1810862116 30782835 PMC6410805

[41] Liu M, Yang S, Bu Y, Zhang N. Female early-career scientists have conducted less interdisciplinary research in the past six decades: evidence from doctoral theses. Humanities and Social Sciences Communications. 2023;10(1):1–16. doi: 10.1057/s41599-023-02392-5

[42] Lindahl J, Colliander C, Danell R. Early career performance and its correlation with gender and publication output during doctoral education. Scientometrics. 2020;122(1):309–330. doi: 10.1007/s11192-019-03262-1

[43] Berggren Å, Almlöv C, D’Urso A, Grubbström A. “Screwed from the start”: How women perceive opportunities and barriers for building a successful research career. In: Frontiers in Education. vol. 7. Frontiers Media SA; 2022. p. 809661.

[44] Misra J, Smith-Doerr L, Dasgupta N, Weaver G, Normanly J. Collaboration and gender equity among academic scientists. Social sciences. 2017;6(1):25. doi: 10.3390/socsci6010025

[45] van der Wal JE, Thorogood R, Horrocks NP. Collaboration enhances career progression in academic science, especially for female researchers. Proceedings of the Royal Society B. 2021;288(1958):20210219. doi: 10.1098/rspb.2021.0219 34493075 PMC8424303

[46] Tartari V, Salter A. The engagement gap:: Exploring gender differences in University–Industry collaboration activities. Research Policy. 2015;44(6):1176–1191. doi: 10.1016/j.respol.2015.01.014

[47] Bozeman B, Gaughan M. How do men and women differ in research collaborations? An analysis of the collaborative motives and strategies of academic researchers. Research policy. 2011;40(10):1393–1402. doi: 10.1016/j.respol.2011.07.002

[48] Abramo G, D’Angelo CA, Murgia G. Gender differences in research collaboration. Journal of Informetrics. 2013;7(4):811–822. doi: 10.1016/j.joi.2013.07.002

[49] Kwiek M, Roszka W. Gender disparities in international research collaboration: A study of 25,000 university professors. Journal of Economic Surveys. 2021;35(5):1344–1380. doi: 10.1111/joes.12395

[50] Bear JB, Woolley AW. The role of gender in team collaboration and performance. Interdisciplinary science reviews. 2011;36(2):146–153. doi: 10.1179/030801811X13013181961473

[51] Meng Y. Gender distinctions in patenting: Does nanotechnology make a difference? Scientometrics. 2018;114(3):971–992. doi: 10.1007/s11192-017-2607-4

[52] Opinions on Deepening the Reform of the Personnel Distribution System in Higher Education Institutions. Ministry of Education of the People’s Republic of China; 1999.

[53] Implementation Opinions on Deepening the Reform of the Personnel System in Higher Education Institutions. Ministry of Education of the People’s Republic of China; Ministry of Personnel of the People’s Republic of China; Organization Department of the CPC Central Committee; 2000.

[54] Shu F, Quan W, Chen B, Qiu J, Sugimoto CR, Larivière V. The role of Web of Science publications in China’s tenure system. Scientometrics. 2020;122:1683–1695. doi: 10.1007/s11192-019-03339-x

[55] Tian M, Lu G. What price the building of world-class universities? Academic pressure faced by young lecturers at a research-centered University in China. Teaching in Higher Education. 2017;22(8):957–974. doi: 10.1080/13562517.2017.1319814

[56] Yang X, Cai X, Li T. Does the tenure track influence academic research? An empirical study of faculty members in China. Studies in Higher Education. 2024;49(3):476–492. doi: 10.1080/03075079.2023.2238767

[57] Qiu J. Publish or perish in China: the pressure to rack up publications in high-impact journals could encourage misconduct, some say. Nature. 2010;463(7278):142–144. 10.1038/463142a 20075887

[58] Yu Y, YL L. Achievements and Reflections on the Development of Women’s Higher Education in China over the Past 70 Years. Women of China. 2019;22:4.

[59] Monitoring Report on the “China Women’s Development Outline (2021-2030). National Bureau of Statistics of China; 2022.

[60] Blue Book of Chinese Overseas Students Development Report (2022). Center for China and Globalization, Bank of China; 2022.

[61] China Education Statistics Yearbook 2016 (Chinese-English). Development Planning Department, Ministry of Education of the People’s Republic of China; 2017.

[62] Brave to be yourself—Interview with biologist Yan Ning; 2024. Available from: https://news.un.org/zh/story/2024/03/1077782.

[63] Wang L. Disappearing Women: A Survey and Analysis Report on Gender Issues in Domestic Academic Institutions; 2016. Available from: https://chinadigitaltimes.net/chinese/423589.html.

[64] Wu X, Ye H, He GG. Fertility decline and women’s status improvement in China. Chinese Sociological Review. 2014;46(3):3–25. doi: 10.2753/CSA2162-0555460301

[65] Liu Y. Women rising as half of the sky? An empirical study on women from the one-child generation and their higher education participation in contemporary China. Higher education. 2017;74(6):963–978. doi: 10.1007/s10734-016-0102-0

[66] Bao L, Wang G. “I am willing to do both well”: Chinese academic mothers facing tension in family and career. Frontiers in Psychology. 2022;13:973110. doi: 10.3389/fpsyg.2022.973110 36405140 PMC9669762

[67] Li B, Shen Y. Publication or pregnancy? Employment contracts and childbearing of women academics in China. Studies in Higher Education. 2022;47(4):875–887. doi: 10.1080/03075079.2020.1817888

