# Supplementary material for: Gender differences, academic patenting, and tenure-track reform in China: Evidence from life sciences at elite universities
Source: PLoS One. 2024 Jul 16;19(7):e0307165. doi: 10.1371/journal.pone.0307165 (PMC11251593; doi:10.1371/journal.pone.0307165)
Supplement: S1 Data — (DOCX) [file pone.0307165.s001.docx]

Zhang, Xin. patent and gender - Chinese universities. Ann Arbor, MI: Inter-university Consortium for Political and Social Research [distributor], 2024-05-15. https://doi.org/10.3886/E202841V1
